# Reference interval for glycated hemoglobin (HbA1c) in non-pregnant, non-diabetic women in Ethiopia: a cross-sectional study

**DOI:** 10.11604/pamj.2025.52.103.49325

**Published:** 2025-11-11

**Authors:** Kassahun Tekle Takiso, Abebaye Aragaw Lemine, Aster Abebe Tsegaye, Mekoya Mengistu Dabulo, Abenet Desalegn W/Senbet

**Affiliations:** 1 Department of Medical Physiology, Addis Ababa University, Addis Ababa, Ethiopia

**Keywords:** Ethiopia, reference values, non-pregnant women, HbA1c, age

## Abstract

**Introduction:**

glycated hemoglobin (HbA1c) serves as a key indicator of average blood glucose levels over the preceding 2-3 months, reflecting cumulative glucose exposure based on erythrocyte lifespan. Research has documented racial and ethnic disparities in the relationship between HbA1c levels and blood glucose. In this case, the non-Hispanic Black individuals consistently exhibit higher HbA1c levels than their non-Hispanic White and Hispanic counterparts. The primary objective of this study was to establish a country-specific reference interval for hemoglobin A1C in non-diabetic, non-pregnant women and to investigate the correlation between HbA1c levels and demographic and clinical characteristics.

**Methods:**

an institution-based cross-sectional study was conducted on 129 non-pregnant, non-diabetic women aged ≥ 18 years in Addis Ababa, Ethiopia (December 4, 2024, to February 28, 2025); data were analyzed using IBM SPSS Statistics. Normality of continuous variables was assessed with Shapiro-Wilk and Kolmogorov-Smirnov tests. The reference interval for HbA1c was calculated parametrically (mean ± 2SD) if normally distributed or non-parametrically (2.5^th^- 97.5^th^ percentiles) if not, other non-normal variables were summarized as median (range). Correlation between HbA1c and demographic or clinical characteristics was assessed using Pearson´s correlation coefficient. Outliers were identified and excluded using Tukey´s method.

**Results:**

the HbA1c values, measured in mmol/mol, were normally distributed with a mean (±SD) of 38(±3.1), yielding a 95% reference interval of 32 - 44 mmol/mol. The corresponding DCCT percentage values ranged from 5.1% to 6.2%, with a mean (±SD) of 5.6% (±0.28). A statistically significant but weak positive correlation was observed between age and HbA1c levels (r =0.284, p = 0.007).

**Conclusion:**

this study found that the lower limit of the normal HbA1c reference interval in Ethiopian women was higher than reported in some previous studies. Age showed a modest influence on HbA1c levels, highlighting the importance of establishing population-specific reference intervals for accurate clinical interpretations.

## Introduction

Glycated hemoglobin (HbA1c) is a critical blood marker that reflects a person´s average blood glucose levels over the preceding two to three months. It is a cornerstone for diagnosing diabetes mellitus and monitoring long-term glycemic control in patients [1,2]. To interpret any diagnostic test like HbA1c, clinicians rely on a reference interval (RI) - the range of values found in a healthy population. This interval is a fundamental tool for distinguishing healthy from disease [3]. International guidelines, such as those from the International Federation of Clinical Chemistry (IFCC), strongly recommend that each laboratory or region establish its own RIs tailored to its specific population [4,5]. This is because RIs are not universal; they can vary significantly due to differences in genetics, ethnicity, diet, race, age, and environmental factors [6-10].

Despite this recommendation, most clinical laboratories find it challenging to establish local RIs due to the high cost, the complexity of recruiting a sufficient number of healthy reference individuals, and the extensive testing required [11]. Consequently, they often rely on manufacturer-based reference intervals (MBRIs), which are convenient but have major limitations. MBRIs may lack generalizability and can be inaccurate for populations not represented in the manufacturer´s original reference study. In contrast, laboratory-based reference intervals (LBRIs), though resource-intensive to develop, provide higher-quality, population-specific data that lead to more accurate diagnosis and better clinical decisions [12,13].

The need for population-specific RIs is particularly evident for HbA1c. Numerous studies have identified ethnic and racial variation in HbA1c levels. For example, at similar blood glucose levels, non-Hispanic Black individuals have been shown to have higher HbA1c levels than non-Hispanic White individuals [14]. Similarly, New Zealand uses a different HbA1c threshold for diagnosing gestational diabetes for women of Asian ethnicity compared to European ethnicity [15], underscoring that a single standard is not appropriate for all. In Ethiopia, there is a lack of locally established reference intervals for HbA1c. Current clinical practice relies on intervals derived from Western populations, which may not be applicable to Ethiopians. This gap increases the risk of misdiagnosis, including both overdiagnosis, leading to unnecessary treatment, and underdiagnosis, resulting in a missed opportunity for timely intervention. Therefore, this study aimed to establish a robust, country-specific reference interval for HbA1c in healthy, non-pregnant, non-diabetic Ethiopian women and to investigate the correlation between HbA1c levels and demographic and clinical variables.

## Methods

**Study design, setting, and period:** an institution-and laboratory-based cross-sectional study was conducted from December 4, 2024, to February 28, 2025, at Janmeda and Teklehaymanot Health Centers in Addis Ababa, Ethiopia. A total of 129 non-pregnant, non-diabetic women aged 18 to 48 years were included in the study. Participant recruitment and sampling were conducted at these two government health centers.

**Ethical consideration:** the study protocol was reviewed and approved by the Institutional Research Ethics Review Committee (IRERC) of the College of Health Sciences, Addis Ababa University (protocol number 014/24/physio). The study was conducted in strict accordance with the ethical principles of the Declaration of Helsinki [16]. Before any data collection, written informed consent was obtained from every participant after the nature and purpose of the study had been fully explained. To ensure confidentiality, all participant data were de-identified using unique study codes. Completed questionnaires and consent forms were stored separately in a locked cabinet, and access to all study materials was restricted to the principal investigator and supervising authors.

**Study population:** the source population comprised non-pregnant, non-diabetic women aged 18 years and above attending routine medical check-ups at the selected health centers in Addis Ababa, Ethiopia.

**Sampling technique:** participants were recruited using a systematic random sampling technique. The sampling frame was obtained from the health center records. A random starting point was chosen by drawing a number between 1 and 9, and every k-th woman was subsequently invited until the required sample size (minimum 120) was achieved, ensuring adequate statistical power for a non-parametric test.

**Sample size:** the sample size was determined based on recommendations from the International Federation of Clinical Chemistry (IFCC), which advises a minimum of 120 individuals for establishing reference intervals using non-parametric methods [17,18]. Our study enrolled 129 participants, thereby meeting and exceeding this requirement. Parametric methods were employed following data normality; the sample size is also more than sufficient to invoke the Central Limit Theorem. This theorem ensures the robustness of parametric test with large sample size (a common threshold being *n* ≥ 30), further validating our analytical approach [19,20].

### Data collection and personnel

**Data collection team:** data were collected by four trained medical professionals-two midwife nurses and two medical laboratory technologists- who were employed at the respective health centers. The entire data collection process was supervised daily by the study authors.

**Training of data collectors:** the data collectors underwent a one-day training session on standardized study procedures. The training covered protocols for participant recruitment based on inclusion criteria, obtaining informed consent, and assigning a unique identification number (ID) to each participant. It also included instructions on administering the questionnaire and ensuring all responses were correctly linked to the participant´s ID. Additionally, the training addressed laboratory procedures, including collecting blood samples into EDTA and SST tubes labeled with the participant IDs, performing immediate point-of-care hemoglobin and random blood sugar tests, and preparing EDTA samples for daily transport to the Black Lion Hospital Laboratory.

### Data collection

**Participant recruitment:** the study enrolled non-pregnant, non-diabetic women aged ≥ 18 years who attended routine medical check-ups at the selected health centers. Eligible participants met the following inclusion criteria: non-pregnant women (confirmed by a negative urine hCG rapid test), age ≥ 18 years, with a random blood glucose (RBS) level < 100 mg/dL and hemoglobin concentration >11 g/dL, in accordance with WHO Standards [21]. Women were excluded if they had a history of diabetes, alcoholism, pregnancy, anemia, or any chronic systemic disease (e.g., hypertension, splenectomy, or hemolytic, hepatic, immunological, renal, or cardiac disease).

### Data collection procedures

**Sociodemographic and clinical characteristics:** after obtaining informed consent, data were collected using a semi-structured questionnaire adapted from similar sources [22-24]. The questionnaire was translated into Amharic via a forward-backward translation process and pilot-tested for clarity and cultural appropriateness. Minor modifications were made to phrasing based on this feedback. The final tool collected data on socio-demographics, clinical characteristics, lifestyle, and health-related factors.

**Laboratory procedures:** biological samples were collected from each participant. A total of 5 mL of whole blood was drawn: 3 mL was dispensed into a K2EDTA tube for hemoglobin and HbA1c measurement, and 2 mL into a serum separate tube (SST) for random blood glucose measurement and serological testing. In addition, approximately 4 mL of urine was collected in a sterile container for pregnancy testing. After phlebotomy, standardized protocols were followed to ensure sample integrity. At the health centers, EDTA whole blood samples were used immediately for hemoglobin and blood group analysis within 40 minutes of collection. Concurrently, SST blood samples were allowed to clot at room temperature for 30 minutes, and then centrifuged at 3000 x g for 10 minutes. The separated serum was immediately analyzed on-site for RBS and other serological tests, while urine hCG testing was also performed on-site. The remaining EDTA whole blood samples were transported to the Black Lion Hospital Laboratory for HbA1c analysis. Black Lion Hospital, the largest national referral hospital in Ethiopia, also serves as a primary training institution for health professionals [25]. HbA1c measurement was performed within 4 hours of collection, ensuring the entire process was completed within a strict 4-8 hour window.

### Biochemical analysis

**All biochemical analyses were performed following standard operating protocols:** HbA1c was quantified using a cobas C 311 analyzer (Roche Diagnostics, Switzerland) via a turbidimetric inhibition immunoassay (TINIA). The analyzer was calibrated according to the IFCC reference system, and two levels of quality controls (Precicontrol Norm and Path) were run daily to ensure accuracy. Plasma glucose was measured using a BS-200 clinical chemistry analyzer (Mindray, China) with the hexokinase method. Two levels of controls (low and high) were analyzed prior to sample testing. Hemoglobin concentration was determined using a Sysmex XS-500i hematology analyzer (Sysmex Corporation, Japan) employing the modified, cyanide-free, sodium lauryl sulfate (SLS)-hemoglobin method.

**Statistical analysis:** data were analyzed using SPSS version 27 (IBM Corp., USA). Potential outliers in all continuous variables were identified and excluded using Tukey´s statistical methods [26]. The normality of continuous variables, including HbA1c, was assessed using the Shapiro-Wilk test and complemented by visual inspection of histograms and Q-Q plots. For variables that satisfied the assumption of normality, reference intervals were calculated parametrically as the mean ± 2 x standard deviation (SD). Continuous variables that did not follow a normal distribution were summarized using the median with range (minimum-maximum). The relationship between HbA1c and demographic and clinical characteristics was assessed using Pearson´s correlation coefficient, selected because: (1) HbA1c demonstrated normal distribution after outlier removal, and (2) Pearson´s correlation is robust to mild deviations from normality with adequate sample size.

## Results

**Figure 1:**
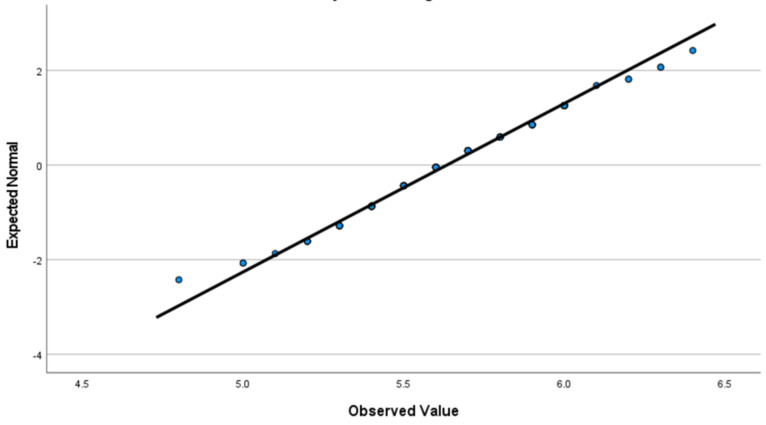
quantile-quantile (Q-Q) plot of HbA1c values in the study population of Ethiopian women; findings from the study conducted at Janmeda and Teklehaymanot Health Centers, Addis Ababa, Ethiopia, 2025

Out of 210 non-pregnant women assessed, 174 were found eligible, and 129 met the inclusion criteria, while 45 were excluded for various reasons: 21 had random plasma sugar levels exceeding 100mg/dl, 4 were diagnosed with anemia, 5 tested positive for hCG, 4 were positive for HBsAg, 1 was positive for HCV, and 5 had other medical conditions. The distribution of HbA1c values was assessed before calculating the reference interval. The Shapiro-Wilk test indicated that HbA1c closely followed a normal distribution (W = 0.981, P = 0.071). Visual inspection of the normal Q-Q plot (Figure 1) further supported this finding, as the observed data points aligned closely with the expected diagonal reference line. Based on the conformation of normality, the reference interval for HbA1c was estimated using the parametric method (mean ± 2 x SD). Continuous variables that did not meet normality assumptions were summarized using the median with range (minimum-maximum).

**Demographic and clinical characteristics:** the baseline demographic and clinical characteristics of the 129 non-pregnant women included in the study are summarized in Table 1. Participants were young to middle-aged women, with a median age of 30 years (range: 18-48 years) and a median body mass index (BMI) of 20.3kg/m^2^ (range: 18.7 - 25.6kg/m^2^). The median hemoglobin (Hb) level was 14.6 g/dL (range: 12-16.4 g/dL). Median systolic and diastolic blood pressures were 116 mmHg (range: 90 -135) and 78 mmHg (range: 70 - 88 mmHg), respectively. The median Random Plasma Glucose (RPG) level was 5.1mmol/L (range: 4.3- 5.5mmol/L). Blood group “O” was the most prevalent (41.9%), followed by groups “A” (30.2%) and “B” (23.3%). All participants tested negative for hCG, hepatitis B, and hepatitis C, and none reported a family history of diabetes mellitus (DM) or gestational diabetes mellitus (GDM).

**Table 1:** baseline demographic and clinical characteristics of non-pregnant women recruited at Janmeda and Teklehaymanot Health Centers, Addis Ababa, Ethiopia from December 4, 2024 to February 28, 2025 (n=129)

Characteristic	Value
**Demographic**	
Age (years)	30.0 (18-48)
**Clinical measurement**	
Weight (Kg)	51.5 (45-75)
BMI, in kg/m^2^	20.3 (18.7-25.6)
Hb (g/dl)	14.6 (12-16.4)
RPG (mmol/L)	5.1 (4.3-5.5)
BP (mmHg)	116 (90-135)/78 (70-88)
**Blood group, n (%)**	
"A"	39 (30.2)
"B"	30 (23.3)
"AB"	6 (4.7)
"O"	54 (41.9)
**Medical history and serological status**	
Family history of DM	Absent
History of GDM	Absent
HBsAg	Negative
HCV	Negative
Urine hCG	Negative

Data for continuous variables are presented as median (range). Categorical variables are presented as n (%). BP is presented as systolic/diastolic (range). BP, blood pressure; BMI, body mass index; Hb, hemoglobin; RPG, random plasma glucose; DM, diabetes mellitus; HBsAg, hepatitis B surface antigen; HCV, hepatitis C virus; hCG, human chorionic gonadotropin

**Reference intervals for HbA1c:** the reference intervals for HbA1c were established in a cohort of 129, non-diabetic non-pregnant women. The 95% reference interval was 32-44 mmol/mol (IFCC standard) and 5.1-6.2% (DCCT standard). The mean HbA1c values were 38 (SD 3.1) mmol/mol and 5.6 (SD 0.28)%, respectively, both centrally located within the calculated reference intervals (Table 2).

**Table 2:** reference intervals and mean values for HbA1c among study participants, recruited at Janmeda and Teklehaymanot Health Centers, Addis Ababa, Ethiopia, from December 4, 2024, to February 28, 2025 (n=129)

Parameter	Units	95% reference interval	Mean (SD)
HbA1c IFCC	mmol/mol	32-44	38(3.1)
HbA1c derived DCCT	%	5.1-6.2	5.6(0.28)

The 95% reference interval represents the central 95% of the observed value. HbA1c, glycated hemoglobin; IFCC, International Federation of Clinical Chemistry; DCCT, Diabetes Control and Complication Trial; SD, standard deviation

**Correlation of HbA1c with demographic and clinical variables:** the relationship between HbA1c (normally distributed) and key demographic and clinical variables was assessed using Pearson´s Correlation Coefficient. Results are presented in Table 3. Age showed a statistically significant, weak positive correlation with HbA1c (r = 0.285, p = 0.007). No significant correlations were observed between HbA1c and random blood glucose (r = 0.010, p =0.914) or weight (r = 0.048, p = 0.588)

**Table 3:** person correlation analysis of HbA1c with demographic and clinical variables among study participants, recruited at Janmeda and Teklehaymanot Health Centers, Addis Ababa, Ethiopia, from December 4, 2024, to February 28, 2025 (n=129)

Variable	r-value	p-value	Statistical Significance	Interpretation
Age	0.284	0.007	Significant (**)	Weak positive correlation
Random blood sugar	0.010	0.914	Not significant	No meaningful association
Weight	0.048	0.588	Not significant	No meaningful association

Correlation strength was interpreted as follows |r| < 0.3 = weak correlation; 0.3 ≤ |r| < 0.7 = strong. Statistical significance was determined at α = 0.05. ** P < 0.01. R-values, Pearson's correlation coefficient

## Discussion

Age, ethnicity, genetic background, and geographical location significantly influence HbA1c values, as highlighted by numerous studies. As no HbA1c reference intervals had previously been developed in Ethiopia, this study aims to establish a reference interval for HbA1c levels in non-diabetic, non-pregnant women in Ethiopia. Currently, clinical practices in Ethiopia rely on manufacturer-provided reference intervals, which may not accurately reflect the unique characteristics of the local population. Developing a population-specific reference interval can enhance the precision of diabetic management in Ethiopia.

In this study, the reference interval for HbA1c in non-diabetic, non-pregnant women was 5.1% to 6.2%, with a mean of 5.6%. HbA1c values were positively associated with age. While the lower limit of HbA1c value (5.1%) was higher than in some previous studies, the upper limit (6.2%) was aligned with findings from Italy (4.8% to 6.2%) [27], China (4.7% to 6.3%) [28], and Nordic Caucasians (4.1% to 6.4%) [29]. The upper limit in the current study is also consistent with the value (≤ 6.5%) established by the American Diabetes Association (ADA), which further considers a value below 6.0% to be optimal [30]. A study conducted at Althagelvin Area Hospital in Londonderry reported an upper limit of 6.5% [31], which is nearly similar to our result. The mean HbA1c value in our study (5.6%) was also comparable to a study conducted in Sudan, which reported a mean of 5.57% among non-pregnant women [32].

However, the reference interval reported in Sudan was wider (4.7% to 6.7%) [33] than in our study (5.1% to 6.2%). Further studies are required to determine the reason for such variations, as differences in dietary habits, genetic predisposition, environmental influences, and other conditions could contribute to inconsistent findings. In contrast, the mean HbA1c value (5.6%) was higher than in another study reported (3.43%) [34]. This represents a striking contrast, highlighting potential differences in glycemic control and overall metabolic health between these populations. The reference intervals in the current study (5.1% to 6.2%), with the lower limit of 5.1% this value were also higher than in the study conducted in South Asia (4% to 5.7%), Pakistan (3.51% to 4.87%), and Japan (3.51% to 4.87%) [23,35,36]. On a similar note, a study conducted among non-diabetic, non-pregnant women in the United States noted an HbA1c value of 5.26±0.44% [37].

Consistent with the previous studies [38-41], we observed a positive correlation between age and HbA1c values. This indicates that advancing age is associated with higher HbA1c levels, possibly due to physiological changes, alterations in glucose tolerance, red blood cell lifespan, metabolic control, and insulin resistance. However, other studies did not find a positive relationship [34,42], suggesting the need for further research to clarify these discrepancies.

**Limitations:** while this study provides robust reference intervals derived from a well-characterized cohort, the sampling framework-restricted to a single urban center-may limit the generalizability of our findings to Ethiopia´s diverse populations or analogous geographical settings. Regional variation in demographic, nutritional, or environmental factors could influence the extrapolation of these reference values.

## Conclusion

This study establishes a locally derived reference interval for HbA1c among non-diabetic, non-pregnant Ethiopian women in Addis Ababa, determined to be 5.1%-6.2%. Notably, the lower limit of this interval is higher than the 5.0-5.1% commonly cited international guidelines, highlighting that global standards may not be directly applicable to this population. We found no significant correlation between HbA1c and weight, or random blood sugar (RBS). In contrast, age showed a weak but significant positive association with HbA1c, suggesting it may need consideration in clinical assessment. Overall, these findings underscore the clinical importance of using population-specific HbA1c reference intervals for the accurate diagnosis and monitoring of dysglycemia in Ethiopia.

**Recommendation:** based on the findings of this study, the locally derived HbA1c reference interval of 5.1%- 6.2% may be considered when interpreting glycemic status among non-pregnant Ethiopian women. Use of this interval could improve the clinical interpretation of HbA1c results by accounting for population-specific characteristics. In addition, these findings provide preliminary evidence that may inform future revisions of national laboratory reference standards. Further multicenter studies involving diverse Ethiopian populations.

### What is known about this topic

- HbA1c is a Well-established marker used to assess the long-term control of hyperglycemia in individuals with diabetes;
- HbA1c levels can vary significantly between ethnic and racial groups due to genetic, environmental, and hematological factor;
- The lack of population-specific reference intervals can lead to misdiagnosis and mismanagement of diabetes.

### What this study adds

- This study establishes, for the first time, a robust country-specific reference interval for HbA1c in non-diabetic, non-pregnant Ethiopian women, ranging from 5.1% to 6.2%;
- The lower limit (5.1%) of this Ethiopian reference interval is higher than those reported in many other populations;
- The study demonstrates that applying a population-specific HbA1c reference interval is essential for accurate clinical interpretation in Ethiopia, as it helps avoid potential over-diagnosis of diabetes when using international reference ranges.
